# The Foliar Anatomy and Micromorphology of *Cyphostemma hypoleucum* (Vitaceae)

**DOI:** 10.3390/plants12122312

**Published:** 2023-06-14

**Authors:** Unarine Rambau, Yougasphree Naidoo, Channangihalli Thimmegowda Sadashiva, Himansu Baijnath, Yaser Hassan Dewir, Katalin Magyar-Tábori

**Affiliations:** 1 School of Life Sciences, University of KwaZulu-Natal, Westville Campus, Private Bag X54001, Durban 4000, South Africa; 2 Plant Production Department, College of Food and Agriculture Sciences, King Saud University, Riyadh 11451, Saudi Arabia; 3 Research Institute of Nyíregyháza, Institutes for Agricultural Research and Educational Farm (IAREF), University of Debrecen, P.O. Box 12, 4400 Nyíregyháza, Hungary

**Keywords:** idioblasts, indumentum, non-glandular trichomes, pearl glands, raphide crystals

## Abstract

*Cyphostemma hypoleucum* (Harv.) Desc. ex Wild & R.B. Drumm is a perennial climber, indigenous to Southern Africa, and belongs to the Vitaceae. Although there have been many studies of Vitaceae micromorphology, only a few taxa have been described in detail. This study aimed to characterize the micro-morphology of the leaf indumentum and determining its possible functions. Stereo microscope, scanning electron microscope (SEM), and transmission electron microscope (TEM) were used to produce images. Micrographs of stereomicroscopy and SEM showed the presence of non-glandular trichomes. In addition, pearl glands were observed on the abaxial surface using a stereo microscope and SEM. These were characterized by a short stalk and a spherical- shaped head. The density of trichomes decreased on both surfaces of leaves as the leaf expanded. Idioblasts that contained raphide crystals were also detected in tissues. The results obtained from various microscopy techniques confirmed that non-glandular trichomes serve as the main external appendages of the leaves. Additionally, their functions may include serving as a mechanical barrier against environmental factors such as low humidity, intense light, elevated temperatures, as well as herbivory and insect oviposition. Our results may also be added to the existing body of knowledge with regard to microscopic research and taxonomic applications.

## 1. Introduction

Specialized secretory leaf structures varying in morphology, location, and function have been found in vascular plants [1]. These structures may be located on the vegetative surfaces of plants or within the plant body [1] and may have secretory or non-secretory abilities [2]. Vegetative surfaces, such as leaves, of the genus *Cyphostemma* have also been observed to be covered with trichomes that may be unicellular or most frequently multicellular in form, and uniseriate or multiseriate [3]. On the surfaces of most plant organs, there are two major types of trichomes: non-glandular and glandular [4]. Non-glandular trichomes appear in abundance in the plant kingdom [5] and may play a significant role in the protection of the epidermal layer and other glandular trichomes during the different growth stages. Furthermore, the non-glandular trichomes prevent microbial spores from attaching themselves to the leaf surface, thus preventing the germination of various pathogens [6,7]. The non-glandular trichomes morphologically may be unicellular or multicellular structures that can both be multiseriate, biseriate or uniseriate, branched, or unbranched and also occur with tapering or blunt tips [7,8]. On the other hand, the glandular trichomes contain modified cells that secrete secondary metabolites [1,9,10] that can interact with pollinators or pests and are usually stored in the cells or exude onto the plant surface [7]. In addition, trichomes can be characterized as either capitate or peltate [11,12,13]. There are significant interactions between trichomes on plants and environmental factors [14,15], thus, scientific interest in plant trichomes has recently increased due to their functional significance and, most importantly, the economic utilization of some trichome-secreted products such as pharmaceutically significant flavonoids, phenols, and saponins [15,16,17].

*Cyphostemma hypoleucum* (Harv.) Desc. ex Wild & R.B. Drumm is a climbing deciduous herb that reaches approximately 2 m in height. It is characterized by double-barrel stems (thus the name: “double-barrel vine”), which are either densely pubescent or tomentose, with tendrils located opposite to the leaves. In South Africa, leaf extracts of *C. hypoleucum* are popular for various ethno-medicinal uses [18,19]. Most medicinal abilities depend on the presence of glandular trichomes and their products [20,21] therefore, this study may help ethnobotanical studies and the use of this plant for medicinal purposes. Limited literature is available on the foliar and general anatomy of *C. hypoleucum* as most species in the genus have not been fully exploited. In addition to being taxonomically useful in general, the trichomes can play an important role in most biological/medicinal activities of plants as they produce secondary metabolites that serve as antioxidants or antimicrobials. Therefore, this study represents the first detailed report on the morphology and the ultrastructure of the trichomes of *C. hypoleucum* using microscopic techniques. This study aims to describe the foliar anatomy and micromorphology of *C. hypoleucum* with a special reference to its trichome structures and raphide crystals. We supposed these traits to be significant in the taxonomic classification as well as in responses to environmental conditions. Thus, this study also may help to recognize the relationship between the leaf anatomy of *C. hypoleucum* and responses of them to both abiotic and biotic stress.

## 2. Results

### 2.1. Trichome Density and Micromorphology

**Figure 1:**
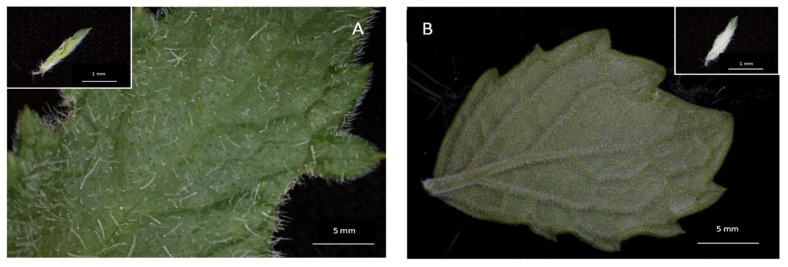
Stereomicrographs of the leaves of *Cyphostemma hypoleucum:* (**A**) Adaxial surface of a young leaf and emergent leaf (inset) showing the distribution of non-glandular trichomes. The distribution of non-glandular trichomes abaxial (**B**) surface of the mature and emergent leaves (inset). Abbreviations: NG = non-glandular trichomes.

**Figure 2:**
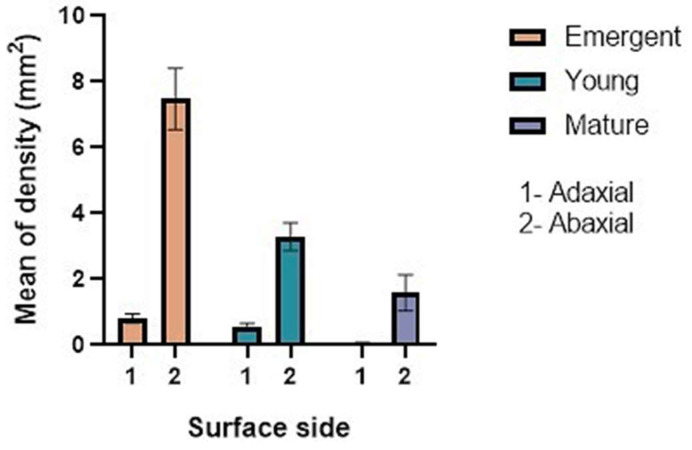
Density of non-glandular trichomes on the adaxial and abaxial surface of leaves of *Cyphostemma hypoleucum* at three stages of development, emergent, young, and mature. Values presented are mean ± SD. All values differ significantly (*p* < 0.05) from each other according to ANOVA.

Stereomicrographs revealed non-glandular trichomes at all stages of development on both adaxial and abaxial surfaces as well as on the margins of leaves (Figure 1A,B). The trichomes become thinner towards the leaf axis (Figure 1A,B). The set of mature non-glandular trichomes was observed to form an indumentum (a dense, hairy covering) on the abaxial surface of mature leaves (Figure 1B). Results obtained through the stereo microscope showed that the abaxial surface of the leaf had a greater density (Figure 2) of trichomes compared to the adaxial surface. The trichomes were found to be abundant, with a distribution across the leaf surface almost completely, giving it a glossy and hairy appearance on the abaxial surface, and their density was greater near the midvein and lateral veins. The emergent and young leaves tended to possess more trichomes per given surface than the mature leaves (Figure 2).

Trichomes of *C. hypoleucum* were found to occur on both leaf surfaces during all three growth stages. Their density varied depending on the leaf surfaces and the leaf growth stages (Figure 2). Results acquired from a one-way analysis of variance (ANOVA) revealed significant differences in the trichome densities of the three growth stages and those of different leaf surfaces. 

### 2.2. Scanning Electron Microscopy of Leaves of C. hypoleucum

**Figure 3:**
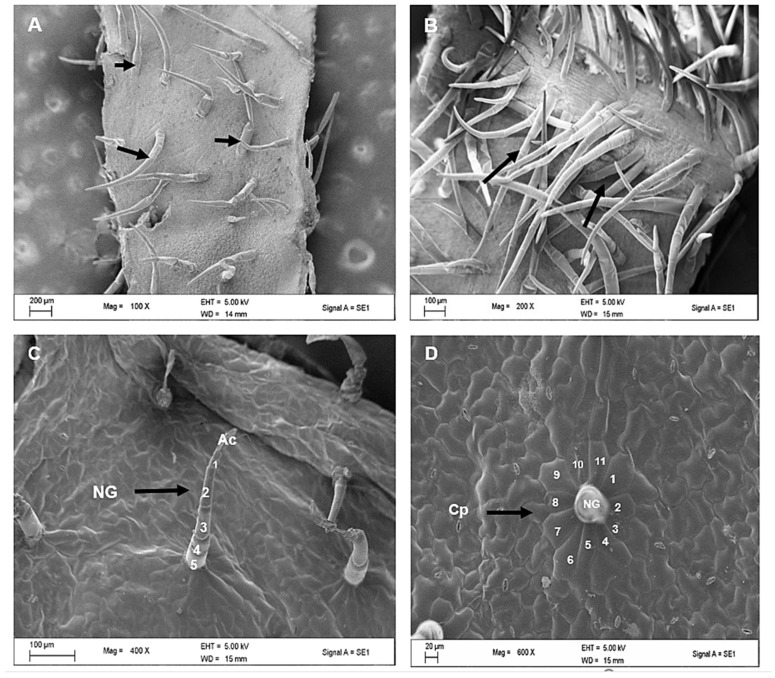
Scanning electron micrographs of *Cyphostemma hypoleucum* leaves showing a dense distribution of non-glandular trichomes (arrows) on the adaxial (**A**) and abaxial (**B**) surfaces. Note a high abundance along the mid-vein. Multicellular, mature non-glandular (indicated by arrow) trichome consisting of about four cells (2–5) and an elongated apical cell as cell 1 (**C**). Cellular pedestal (arrow) in a rosette arrangement consisting of eleven cells (1–11) around a non-glandular trichome (**D**). Abbreviations: NG–non-glandular trichome; Ac–apical cell; Cp–cellular pedestal.

Figure 3 shows electron micrographs of the appearance of non-glandular trichomes on both the adaxial and abaxial surfaces of the leaves. Trichomes were concentrated along the midvein on the abaxial surface (Figure 3B), as previously noted in the stereomicrographs (Figure 1B). The long-sized non-glandular trichome filament consisted of about 3–5 cells and an elongated apical cell (Figure 3C). Figure 3D shows the wide base attached to the epidermal layer of the non-glandular mature trichome being surrounded by special additional cells that stand out from other epidermal cells known as a cellular pedestal. The cellular pedestal was a buildup of an assembly of five to eight epidermal cells that formed a rosette around the base of the trichome. 

**Figure 4:**
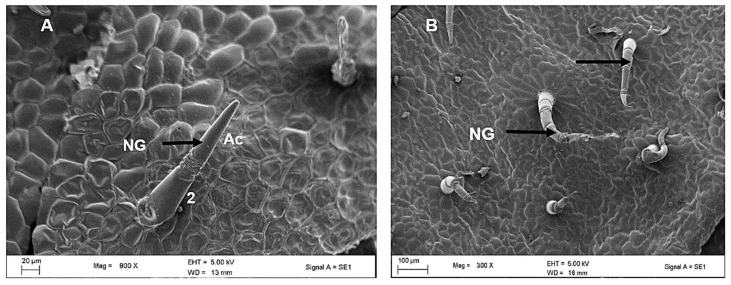
Scanning electron micrographs of trichomes in *C. hypoleucum* leaves. The short non-glandular trichome consisting of a single cell (2) and an elongated apical cell (**A**); and a long non-glandular trichomes (arrows) consisting of more than two cells bent towards the epidermis (**B**). Abbreviations: NG–non-glandular trichome; AC–apical cell.

**Figure 5:**
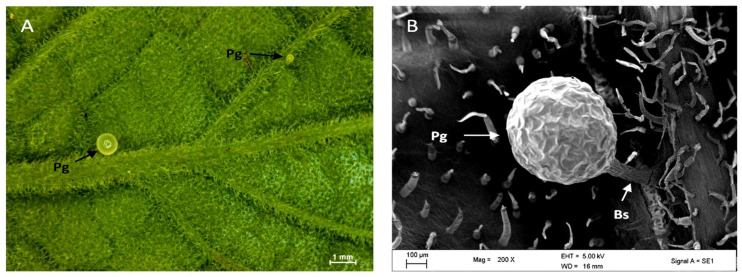
Stereo-micrograph showing the appearance of a pearl gland on the abaxial surface of the *C. hypoleucum* leaf (**A**). Scanning electron micrograph showing the morphology of the pearl gland and base stalk (**B**). Abbreviations: Pg–pearl gland; Bs–base stalk.

Trichomes of two sizes were found: long and short, the latter consisting of 1 or 2 cells which were observed to be parallel to the epidermis (Figure 4A) whereas the former were perpendicular to the typical epidermis (Figure 4B). Pearl glands or food bodies were also observed along midveins and lateral veins on the abaxial surface of the leaf with stereo microscope (Figure 4A) and scanning electron microscope (Figure 4B). The pearl glands in *C. hypoleucum* were observed to be globose, translucent (Figure 5A) and have a uniseriate epidermis (Figure 5B), with a short pedicel attaching them to the epidermis, when observed under stereomicroscopy and SEM, respectively.

### 2.3. Leaf Anatomy Based on Light and Transmission Electron Microscopy (TEM)

**Figure 6:**
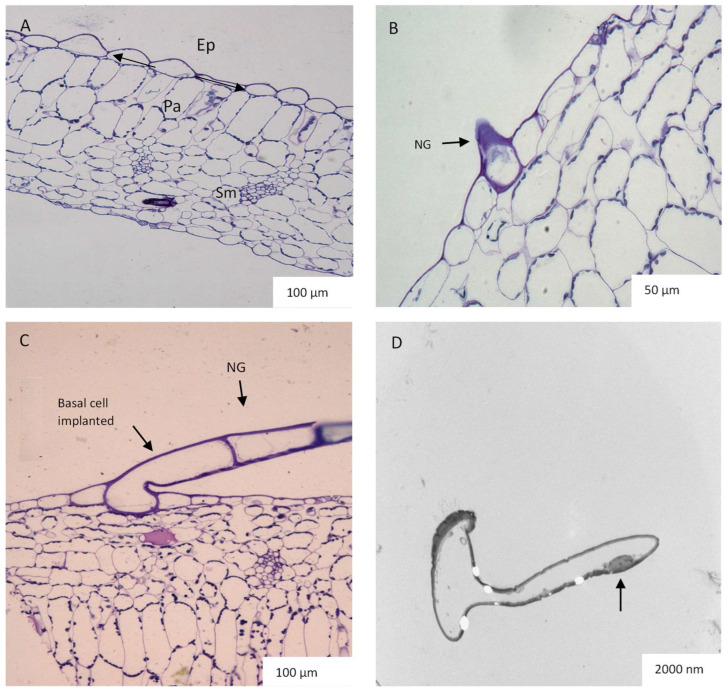
Light micrograph of *C. hypoleucum* leaf showing layers of epidermal cells, the palisade layer, and spongy mesophyll (**A**). Light micrograph of the transverse section of the leaf showing a small protrusion on the leaf surface of a developing trichome (**B**). Micrograph showing a transverse section of leaf with a well-developed non-glandular trichome arising from the leaf epidermis (**C**). Transmission electron micrograph showing a highly vacuolated non-glandular trichome with a nucleus (arrow) located in the peripheral cytoplasm of the trichome (**D**). Abbreviations: Ep–epidermal cells; Pa–palisade layer; Sm–spongy mesophyll; NG–non-glandular trichome.

**Figure 7:**
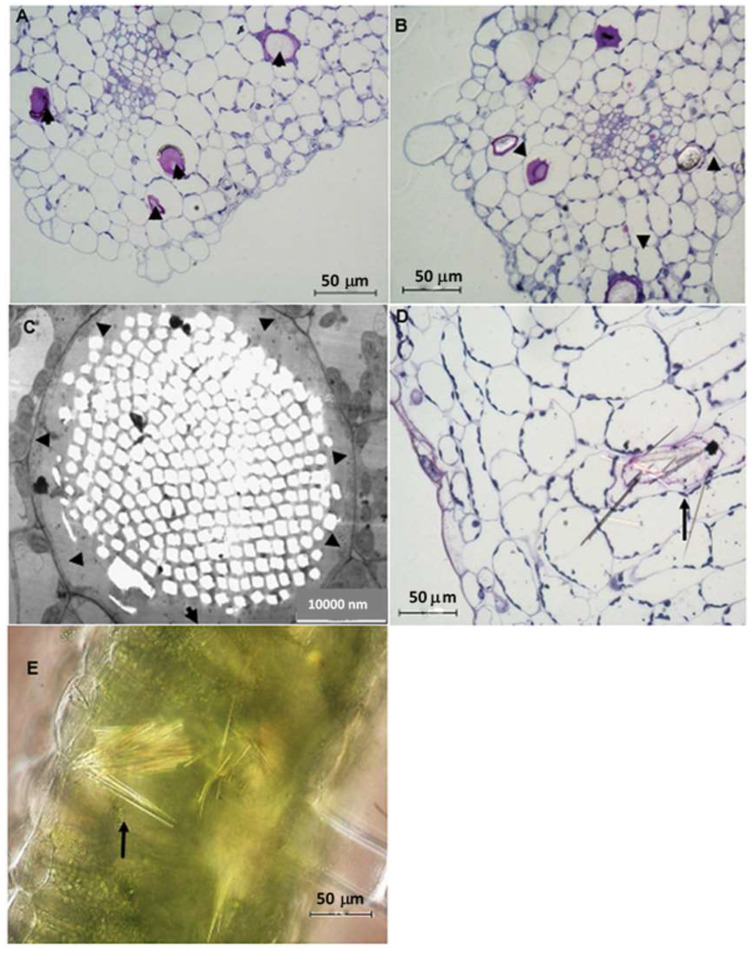
Young (**A**) and mature (**B**) leaf mesophyll of *C. hypoleucum* containing idioblasts (arrows) with calcium oxalate crystals. TEM micrograph showing a mature raphide bundle pushing the cytoplasm against the sides of the cell (black arrows showing raphide crystals) (**C**). Note the dotted white areas within the idioblasts wherein crystals were accumulated. Light micrograph showing needle-like crystals (arrow), ejected from an idioblast (**D**). Light micrograph of raphide crystals taken with a polarizing microscope, they closely packed indicated birefringence under polarized light (**E**).

In transverse sections, it is observed that the epidermis is made up of a single layer of cells while the palisade tissue consists of about one or two layers of densely arranged cells. The spongy mesophyll has loosely organized cells that are located toward the abaxial surface (Figure 6A). Small protrusions were observed on both surfaces of the leaf (Figure 6B), which later developed and elongated to form non-glandular trichomes (Figure 6C). Figure 6D shows the TEM of a highly vacuolated non-glandular trichome with dense material located towards the trichome head. In addition, a hollow area was detected inside the trichome as validation of the large vacuoles in the trichomes of *C. hypoleucum*. In the transverse sections viewed by light microscopy, enlarged idioblasts containing calcium oxalate crystals were visible which were widely distributed in the palisade parenchyma (Figure 7A–C). The cytoplasm is often pushed against the sides of the cell wall in the idioblast (Figure 7C). TEM micrographs show small holes occupied by crystals (Figure 7C). Ejected needle-like crystals also can be seen (Figure 7D).

## 3. Discussion

### 3.1. Trichome Density and Morphology

This is a comprehensive study of the micromorphology of the foliar indumentum and underlying mesophyll, which also covers the usefulness of indumentum characters in taxonomic differentiation. According to the glossary of Payne [11], the trichomes of *C. hypoleucum* were described as non-glandular. Both the adaxial and abaxial epidermis were found to be largely covered in non-glandular trichomes projecting above the leaf surface. However, only a single specific type of trichome was observed. The presence of diverse types of non-glandular trichomes is a frequent characteristic across Vitaceae. The grape plant (Vitis vinifera L.) was also observed to have only non-glandular trichomes, which were found to be filamentous, with a prostrate or erect orientation [22,23]. According to the general description of the physical characteristics of non-glandular trichomes [24], it could be confirmed that *C. hypoleucum* trichomes are uni- or multicellular hairs with an extended apical cell. The presence of these trichomes on both surfaces gives the leaf a hairy appearance. The density of trichomes over the leaf surface suggests that they might be involved in protecting against small insects and herbivores during the early stages of development [25]. In this study, the overall trichome density on both the adaxial and abaxial surfaces was observed to be higher in emergent leaves, followed by young ones, whereas the mature leaves had a significantly low trichome density. It has been assumed before that as the leaf expands the trichome density diminishes [26], due to the leaf expansion throughout leaf development. This shows that the importance of trichomes might become less as leaves mature and that their function is most probably associated with the earliest stages of leaf development when they densely cover the whole leaf. Similarly, this was observed in this study across the three growth stages (Figure 2). Trichome density has also been assumed to indicate structural acclimation of a plant’s epidermal tissues to low temperatures [26,27]. This results in the trichomes serving as a protective adhesive layer that keeps dewdrops off the leaf surface, avoiding frostbite, thus promoting efficient gaseous exchange throughout the plant’s developmental stages [28,29]. However, the question arises as to whether they respond to environments on an anatomical level.

Recent studies have shown that trichomes may have a more expansive role in plant–environment interaction. The interaction is intimate, as plants rely on pollinators for reproduction and may form a symbiotic relationship with microbes. Additionally, a defense system develops, that molds the plant’s phenotype through evolutionary mechanisms, for survival to take place [30,31]. Trichomes act as a barrier during attacks from insects and small herbivores by limiting their movement and can negatively influence insect oviposition and feeding, thus preventing pest invasion [25,32,33,34]. Furthermore, trichomes are often composed of cellulose and other substances of low nutritional value and are not palatable for insects [35,36]. Mechanical defense might also be influenced by trichome density over the surface of the leaf. Studies have generally focused on the adaxial surfaces of leaves; however, it was suggested in studies carried out on *V. vinifera*, *V. davidii* (Rom. Caill) Föex., and *V. doaniana* Munson [37] that trichomes on the abaxial surface might be as important as the adaxial surfaces as they may provide canopies to shield the leaf surface from destructive ultraviolet rays [38,39,40,41].

Other researchers have observed that abaxial surface trichomes have a greater ability at retaining water [42], which reduces the rate of transpiration and may serve as accumulation sites for heavy metals from the soil, thus improving heavy metal tolerance [43,44,45,46,47,48]. Trichomes also consist of protective tissue that can respond to many environmental cues during its development. This is done by showing a variety of visual and mechanical properties as observed in a few studies [49,50]. 

We observed pearl glands constituting food bodies along the midveins and lateral veins on the abaxial surface of the leaf using a stereo microscope (Figure 5A) and SEM (Figure 5B) as also described by other researchers [51]. They are attached to the leaf epidermis by a short stalk (Figure 5B) that is characterized by a globular appearance (Figure 5A). It has been suggested that their function is to attract ants, thus functioning as a food reward and maintaining an ant-plant mutualistic symbiosis [51,52]. It has also been suggested that pearl glands may play a role in an indirect defense mechanism [53]. While the ants are rewarded with sugar, they keep the herbivores away. However, they may also occur in relation to the vigour of the plant or humidity [53,54]. A positive correlation was proven between the adequate nutritional status of the *C. verticillata* (L.) Nicolson & C.E. Jarvis and the number of pearl glands present on the leaves [51]. Pearl glands have also been observed to appear on plants that occur along the African, American, and Indo-Malaysian tropical and subtropical regions, explaining the humidity dependence of their production [55]. The presence of pearl glands in this study is not unexpected as they often occur in members of the family Vitaceae [51]. Pearl glands have been observed on the abaxial surfaces of plants belonging to Vitaceae such as *V. vinifera* [56] and *C. verticillata* [57]. The transverse sections of *C. hypoleucum* leaves show a dissimilarity between the structure of the tissue on both the adaxial and abaxial surfaces. The epidermis is made up of a single layer while the palisade tissue is about one or two layers of closely arranged cells packed with chloroplasts, playing a significant role in photosynthesis [58]. The spongy mesophyll has loosely organized cells to facilitate gaseous exchange [58] and distributes towards the abaxial surface (Figure 6A). In the transverse section of the leaf, there appear small protrusions on both surfaces (Figure 6B) with some epidermal cells elongating to form the non-glandular trichomes (Figure 6C). Non-glandular trichomes seem to arise from a series of periclinal divisions which have also been observed in a majority of flowering plants [6,17].

### 3.2. Idioblast Crystals

Idioblasts with raphides differing in size or length are sparsely distributed throughout the leaf tissue. These structures were found in all three growth stages in the palisade cells of mesophyll (Figure 7A–C). The idioblasts with raphides are visibly bigger than the neighbouring cells, making them conspicuous. As new idioblasts begin to form, the distance between the idioblasts decreases, suggesting that each idioblast is strategically positioned to accumulate calcium oxalate crystals in a specific area of plant tissue, as shown in Figure 7A,B. Several members of the Vitaceae have been observed to contain a diversity of calcium oxalate crystals with raphides being the more common type [24,56,57,59,60]. Most plants do not have a well-defined excretory system suited for the disposal of excess calcium, as such, it was hypothesized that the calcium present in the crystals might be sequestrated into the raphide bundle from the surrounding cells [61,62,63]. However, it has been hypothesized and confirmed that most of the calcium found in raphides is absorbed from calcium-rich soils [64]. This phenomenon was modelled by placing a plant in a concentrated calcium solution and it was observed that the number of idioblasts formed in the plant tissues increased when calcium was present in the solution at a high concentration [64,65,66]. The idioblasts containing the raphides are elongated, almost resembling a cylindrical tube, and contain acicular crystals in a chamber referred to as a “Metcalfe and Chalk bag” [24]. Previous studies have provided evidence supporting the hypothesis that needle-shaped crystals provide an efficient protective role against herbivores, as they are released into the mouth during herbivory [58,67,68]. The needle-shaped raphides in *C. hypoleucum* (Figure 7D), have been observed to occur in large numbers in flowering plants, including the leaves of *Cissus verticillata* [57]. The type and shape of the crystals found in *C. hypoleucum* are similar to the same type observed for *Vitis* (Type IV) crystals, indicating the significance of crystals for taxonomic classification [62,69]. The two chemical forms of calcium oxalate crystals, monohydrate, and dihydrate, are both found in plants [69]. Under polarized light (Figure 7E) the crystals in *C. hypoleucum* showed high birefringence, indicating that they are monohydrate, which is a common occurrence in most plant species [70]. The purpose of raphide containing idioblasts is poorly understood, although numerous roles such as mechanical intervention have been proposed [71,72]. The presence of calcium oxalate crystals in edible food plants such as spinach, berries, yams, and the commonly used red leaf herbs (Amaranthus hybridus L.) [64] has been reported to have beneficial as well as harmful effects on human health [73,74,75,76]. It is not known whether calcium oxalate crystals confer medicinal potential, however, in *Cissus quadrangularis* L. they were suggested to play an important role in ayurvedic medicine preparation used for abrasions and fractures [77]. Results from the EDX analysis showed the presence of similar trace elements in *Vitis vinifera* leaves, suggesting that although calcium oxalate crystals occur in members of the Vitaceae family, they may also have a therapeutic effect [56].

## 4. Materials and Methods

### 4.1. Plant Collection

Fresh leaflets of *C. hypoleucum* were collected from the University of KwaZulu Natal Westville Campus (29.8178° S, 30.9427° E), in the spring of November 2015 for the study of trichomes. Voucher specimens (Rambau and Baijnath 1) were prepared and deposited at the Ward Herbarium (UDW), School of Life Sciences at the University of KwaZulu-Natal, South Africa. To investigate the foliar indumentum and trichome distribution, randomly selected leaf specimens at three growth stages, emergent (<1 cm), young (1–5 cm), and mature (5–10 cm), were used for each sample preparation batch (n = 20). The sizes of the leaves were used to differentiate age. Thereafter, the leaves were grouped into three sets of adaxial and abaxial samples.

### 4.2. Stereomicroscopy

Entire leaf samples were secured onto glass microscope slides using two-way adhesive tape. Images of trichome distribution on both leaf surfaces were captured using a Nikon DXM1200C color camera fitted onto the Nikon AZ100 stereo microscope.

### 4.3. Scanning Electron Microscopy (SEM)

To investigate trichome distribution and morphology, leaf samples from three growth stages were sectioned around the midrib and quenched in liquid nitrogen (−196 °C), followed by freezing overnight in an Edwards Modulayo freeze-dryer (Edward, Sussex, UK) at −60 °C at a vacuum of 0.1 Torr. The leaf segments were secured onto brass stubs with two-way adhesive carbon conductive tape and sputter coated for 10 min with gold in an argon atmosphere with an SC500 Polaron Sputter Coater unit (Quorum Technologies Ltd., London, UK). The sample segments were then observed with a Zeiss LEO 1450 Scanning Electron Microscope at 5 kV and a working distance of 14–15 mm.

### 4.4. Light Microscopy and Transmission Electron Microscopy (TEM) 

Preparations involved fixing fresh leaf segments (approximately 4 mm^2^) of *C. hypoleucum* for 24 h in 2.5% glutaraldehyde. The leaf material was washed three times for 5 min each with sodium phosphate buffer followed by post-fixation with 0.5% osmium tetroxide for one hour in a dark cupboard to prevent degradation of the compound after exposure to light. The samples were dehydrated twice over five minutes each for the 30%, 50%, and 75% acetone and distilled water concentrations and 10 min for the 100% acetone concentration. After acetone dehydration, the samples were infiltrated using equal parts of acetone and resin for 4 h and then embedded in 100% Spurr’s resin for 8–24 h [78]. The samples were allowed to polymerize for 7 h at 70 °C. To acquire survey sections for Light microscopy, semi-thin (1–2 μm) sections were cut with glass knives using the Reichert Jung Ultracut-E ultramicrotome (Reichert-Jung, Vienna, Austria) [79] and placed on a glass slide after staining with 0.05% toluidine blue and thereafter observed under a Nikon Eclipse 80i light microscope (Nikon. Tokyo, Japan) interfaced with a Nikon DS-Fil camera and NIS-Elements imaging software package (NIS-Elements D 3.00, SP4 (Build 502). The ultra-thin sections for TEM, (60–70 nm thick) were placed onto 100-square-mesh copper grids, allowed to stain for 10 min in 2.5% uranyl acetate, and then rinsed with distilled water, followed by 10 min in Reynold’s lead citrate solution [80] and a distilled water rinse. These sections were observed with the Jeol 1010 TEM (Tokyo, Japan). Images were captured with the Olympus Mega View III CCD Camera (Soft Imaging System GmBH, Münster, Germany) at 100 kV.

### 4.5. Data Analysis

The trichome densities were analyzed using One-way Analysis of Variances (ANOVA), using the statistical software package IBM SPSS Statistics for Windows (Version 24.0, IBM^®^, New York, NY, USA). A *p*-value of <0.05 was acknowledged as corresponding to significant differences. 

## 5. Conclusions

The foliar indumentum and underlying mesophyll of the leaves of *C. hypoleucum* have been characterized for the first time. *Cyphostemma hypoleucum* shares many characteristics of Vitaceae, such as non-glandular trichomes, pearl glands, and raphide crystals. Trichomes on the leaves of *C. hypoleucum* were found to be multicellular, uniseriate, and non-glandular. Pearl glands with a globular shape characteristically appear on the surface of the leaves. The raphide crystals are needle-like and encased in a raphide bundle. The results obtained in this study could potentially benefit future anatomical research for *C. hypoleucum*. Based on the results of *C. hypoleucum* foliar anatomy and micromorphology analysis obtained in this study, further investigation is needed to analyze interactions between *C. hypoleucum* and herbivores or to study the plant × environment interactions.

## Data Availability

The data that support this study will be shared upon reasonable request to the corresponding author.
